# Urinary microbiome profiling as a non-invasive tool for identifying biomarkers in systemic lupus erythematosus and lupus nephritis

**DOI:** 10.3389/fcimb.2024.1364333

**Published:** 2024-12-03

**Authors:** Bo Shi, Fei Chen, Jianmin Gong, Adeel Khan, Xiang Qian, Zhipeng Xu, Ping Yang

**Affiliations:** ^1^ Department of Clinical Laboratory, Nanjing Jiangning District Hospital of Traditional Chinese Medicine (TCM), Nanjing, China; ^2^ Department of Clinical Laboratory, Nanjing Drum Tower Hospital Clinical College of Nanjing Medical University, Nanjing, Jiangsu, China; ^3^ College of Life Science, Yangtze University, Jingzhou, China; ^4^ Department of Biotechnology, University of Science and Technology Bannu, Bannu, KP, Pakistan; ^5^ Department of Laboratory Medicine, The First Affiliated Hospital of Nanjing Medical University, Nanjing, Jiangsu, China; ^6^ Department of Pathogen Biology, Jiangsu Province Key Laboratory of Modern Pathogen Biology, Nanjing Medical University, Nanjing, Jiangsu, China

**Keywords:** systemic lupus erythematosus, lupus nephritis, urinary microbiota, 16S, biomarker

## Abstract

**Introduction:**

Bacteriome alterations have been implicated in the pathogenesis of systemic lupus erythematosus (SLE). However, the relationship between SLE and the urinary microbiome remains underexplored. This study aimed to characterize the urinary microbiome of SLE patients using 16S rRNA sequencing and to investigate its correlations with clinical parameters through integrative analyses.

**Methods:**

Urine sediment samples were collected from individuals with SLE and lupus nephritis (LN) (n = 20), SLE without LN (n = 22), and healthy controls (HCs) (n = 23). DNA was extracted and subjected to 16S rRNA sequencing to profile the urinary microbiome. Receiver operating characteristic (ROC) curve analysis was conducted to evaluate the diagnostic efficacy of urinary microbiota, while Spearman’s correlation analysis was employed to identify links between specific microbial taxa and clinical parameters. Functional predictions of bacterial roles were performed using Picrust2.

**Results:**

The urinary microbiota diagnostic model exhibited excellent performance in distinguishing SLE patients from HCs. Spearman’s analysis revealed significant correlations between the urinary microbiome and clinical parameters. Specifically, *Sphingomonas* and *Lachnospiraceae* genera showed positive correlations with vitamin D levels, cylinderuria, and proteinuria, while *Pedobacter*, *Aquabacterium*, *Delftia*, and *Achromobacter* displayed negative correlations with proteinuria and albumin-to-creatinine ratio (ACR). Functional predictions indicated that the urinary microbiome might influence immune regulation through modulation of signaling pathways and metabolic processes.

**Discussion:**

Our study is the first to reveal dysbiosis in the urinary microbiome of patients with SLE. Certain bacterial taxa in the urinary microbiome were identified as potential diagnostic biomarkers for SLE. Furthermore, the functional implications of these bacterial communities suggest their involvement in immune modulation, highlighting the potential for further investigation into their roles in SLE pathogenesis and diagnosis.

## Introduction

Systemic lupus erythematosus (SLE) has a wide spectrum of clinical and immunological manifestations as an autoimmune disease. Lupus Nephritis (LN) stands out as a prevalent and serious condition with high degree lethality for individuals with SLE. LN haunts a substantial percentage of adults (30-60%) and the majority of children (70%) diagnosed with SLE (Yu et al., 2017; Davidson et al., 2019; Catalina et al., 2020). The varied and unpredictable nature of disease manifestations and fluctuations in disease activity pose significant challenges in accurately diagnosing and effectively managing SLE (Gasparotto et al., 2020). Certainly, the complexity and variability of SLE symptoms suggest that the disease is not a single phenotype, but rather a highly complex and diverse entity (Azzouz et al., 2019). The current diagnosis of SLE primarily depends on clinical manifestations and laboratory tests, including antinuclear antibodies (ANA), anti-double-stranded DNA (anti-dsDNA), and anti-Smith antigen (anti-Sm). However, the sensitivity and specificity of these markers are relatively low. Despite the fact that LN patient’s prognosis have ameliorated over the prior three decades, its treatment remains challenging, and require a comprehensive understanding of the underlying pathogenesis (Anders et al., 2020). Therefore, it is essential for SLE patients to monitor their kidney function and seek appropriate medical care if LN is suspected or diagnosed. The benchmark procedure for detecting LN is renal biopsy (Mejia-Vilet et al., 2022); but the approach is invasive and carry the risk of excessive bleeding that may cause the death of patients (Yang et al., 2021). Additionally, kidney biopsies are not convenient for rheumatologists to continuously monitor disease progression in patients. Hence, novel noninvasive biomarkers for SLE diagnosis, monitoring, and prognosis were urgently needed.

The subject of majority of investigations of SLE has been focused on the gut microbiota while urinary microbiome is rarely explored (Ling et al., 2017; Lundy et al., 2021; Miller et al., 2022). Recent investigations have weakened the customary notion that claims urine is sterile by indicating the presence of varied microbial systems in the urinary system (Ainsworth, 2017). In fact, this community of microorganisms has influence on health of urinary tract and could contribute to the onset and course of certain urological ailments. Updated research has indicated the potential involvement of urinary microbiome both in the etiology of urinary tract infections as well as in the preservation of urinary tract wellness, bladder cancer, and other urinary disorders (Bucevic Popovic et al., 2018; Martin et al., 2022). Additionally, the urinary microbiome has been linked to other physiological processes such as immune function (Liu et al., 2017) and neurological function, highlighting the far-reaching impact of this microbial community on overall health. Therefore, studying the urinary microbiome holds great potential for developing novel diagnostic and therapeutic approaches for a variety of urological conditions. Hai Bi et al. identified that urinary microbiota may serve as a potential biomarker and therapeutic target for bladder cancer (Bi et al., 2019). Jiayang Jin et al. demonstrated that the urinary tract microbiota profile in rheumatoid arthritis (RA) is characterized by increased microbial richness and altered taxonomic composition, which correlates with the disease’s immunological and metabolic changes, highlighting the interaction between urinary microbiota and host autoimmunity (Jin et al., 2023). However, little is known about their function in autoimmune illnesses, specifically in SLE.

In this study, urine specimens samples were analyzed using 16S rRNA gene sequencing to gain detailed insights into the urinary microbiome. The findings suggest a potential role of urinary microbiome dysbiosis in the pathogenesis of SLE.

## Materials and methods

### Attributes of patients and their clinical data

**Figure 1:**
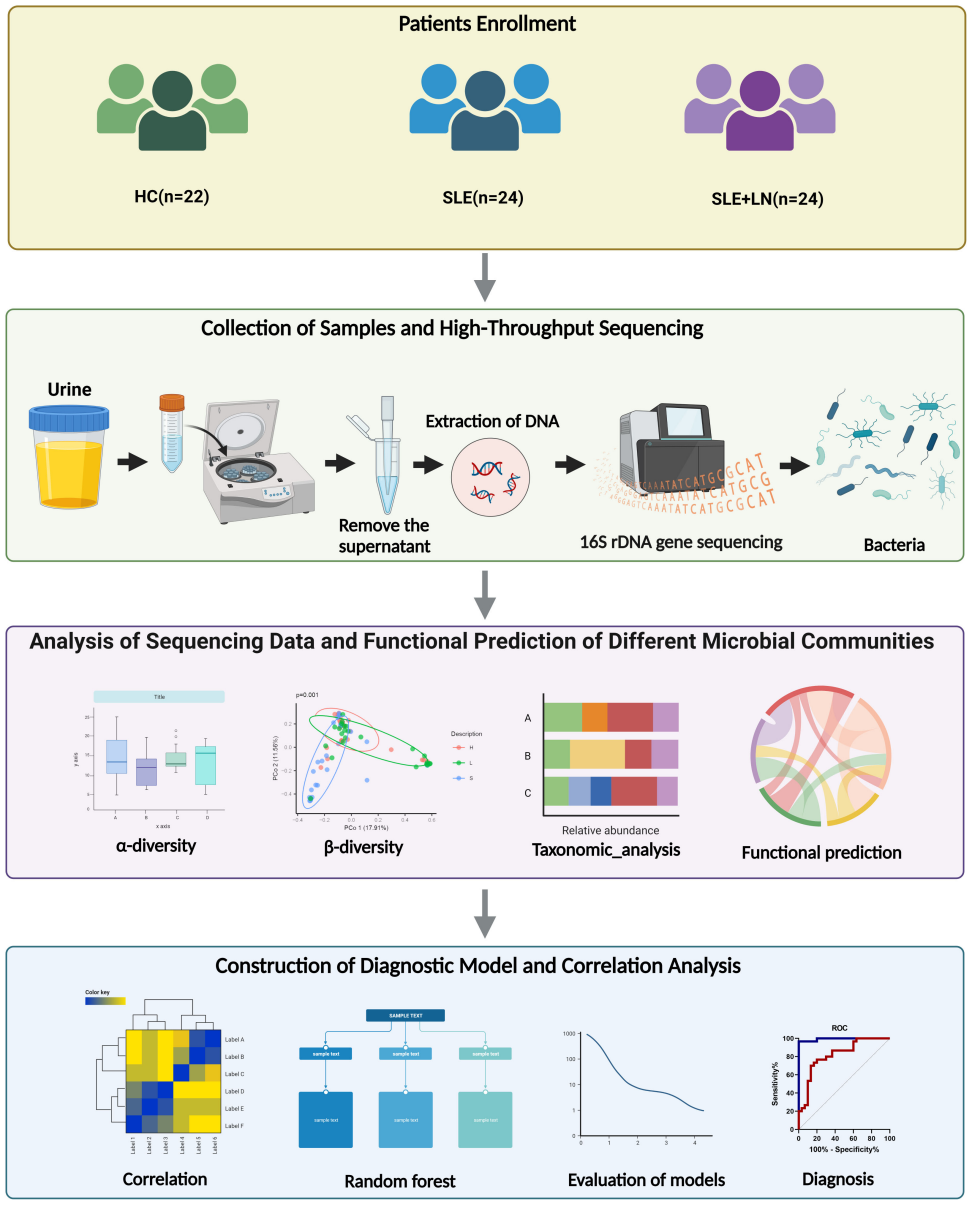
The experimental design flowchart.

We recruited a total of 20 SLE patients with LN, 22 SLE patients without LN, and 23 healthy controls (HCs) for this study in Nanjing Drum Tower Hospital, from February 2022 to September 2022. All enrolled patients were female, and there were no statistically significant differences in age among the three groups. **Figure 1** presents an overall design concept and workflow for the experiment and **Table 1** provides an overview of the clinical attributes of the respective groups. The disease activity of SLE patients was assessed using the SLE Disease Activity Index (SLEDAI) (Bombardier et al., 1992; Hochberg, 1997). The HC group excluded individuals with other diseases such as autoimmune diseases, tumors, diabetes, and urinary tract infections. Informed consent was obtained from all research participants, and the study was approved by the ethics committee of Nanjing Drum Tower Hospital (ID: 2022-466-01).

**Table 1:** Statistics of clinical information of enrolled participants.

clinical characteristic	HCs	SLE without LN	SLE with LN	*P* value (SLE without LN vs SLE with LN)
	23	22	20	
Age, years	41(27-45)	37(26-52)	33(25-44)	0.900
Proteinuria, n (%)	NA	3(14)	20(100)	<0.0001****
Hematuria, n (%)	NA	4(18)	14(70)	<0.001***
pyuria, n (%)	NA	3(14)	11(55)	0.005**
Cylinderuria, n (%)	NA	0(0)	13(65)	<0.001***
24h proteinuria, median (IQR), mg/24h	NA	169(102-201)	1411(450.5-4755.9)	<0.0001****
ACR, median (IQR), mg/g	NA	5.6(5.2-8.3)	875.4(219.2-1910.4)	<0.001***
WBC, median (IQR), ×10^9/L	NA	4.7(3-6.1)	5.3(3.4-7)	0.338
Lymphocytes, (IQR), ×10^9/L	NA	0.9(0.5-1.3)	1.1(0.7-1.6)	0.357
Hb, median (IQR), g/L	NA	113(82-127)	107(93-115)	0.427
PLT, median (IQR), ×10^9/L	NA	199(160-249)	153(112-270)	0.268
ESR, median (IQR), mm/h	NA	28(13-52)	33(24-54)	0.251
Total Protein, median (IQR), g/L	NA	66.1(62.2-73.7)	54.6(49.2-65.1)	<0.001***
blood albumin, median (IQR), g/L	NA	37.6(36.8-40.1)	30.6(27.3-34.2)	<0.001***
globulin, median (IQR), g/L	NA	29.5(24.8-35.4)	25.6(20.6-30.1)	0.049*
A/G, median (IQR)	NA	1.3(1.1-1.6)	1.3(1.1-1.4)	0.632
GLU, median (IQR),mmol/L	NA	4.5(4.1-4.9)	4.4(3.7-4.9)	0.284
Urea nitrogen, median (IQR),mmol/L	NA	4.6(3.9-6.1)	11.4(5-16.8)	<0.001***
Creatinine, median (IQR),umol/L	NA	49(39-54)	66(52-124)	0.004**
Uric acid, median (IQR),umol/L	NA	307(210-332)	374(301-477)	0.004**
Total CO2, median (IQR),mmol/L	NA	24.9(23.4-25.8)	23.7(21-25.6)	0.151
eGFR, median (IQR), ml/min/1.73m^2	NA	128.3(115-189.5)	102.5(41.3-119.5)	0.004**
C3, median (IQR), g/L	NA	0.9(0.6-1)	0.6(0.4-0.9)	0.110
C4, median (IQR), g/L	NA	0.1(0-0.2)	0.1(0-0.2)	0.481
CD3+ T cells (IQR), ×10^9/L	NA	0.7(0.3-1.1)	0.8(0.5-1.1)	0.482
CD3+CD4+ T cells (IQR), ×10^9/L	NA	0.4(0.1-0.7)	0.3(0.2-0.5)	0.871
CD3+CD8+ T cells (IQR), ×10^9/L	NA	0.3(0.1-0.5)	0.5(0.2-0.6)	0.099
B cells (IQR), ×10^9/L	NA	0.1(0-0.2)	0.1(0.1-0.2)	0.646
NK cells (IQR), ×10^9/L	NA	0.1(0-0.1)	0.1(0-0.2)	0.449
Th/Ts, median (IQR)	NA	1(0.6-1.6)	0.7(0.4-1)	0.105
anti-dsDNA, median (IQR)	NA	95.4(11.5-289.6)	264(189.7-422.2)	0.142
ANA, n (%)	NA	15(68)	11(55)	0.238
25-(OH) D3, median (IQR), ng/mL	NA	15.8(10.2-20.2)	16.8(8.2-26.1)	0.904
SLE-DAI, median (IQR)	NA	4(2-5)	11(8-14)	<0.0001****

ACR, albumin-to-creatinine ratio; ESR, erythrocyte sedimentation rate; GLU, glucose; CREA, serum creatinine; eGFR, glomerular filtration rate; C3, complement C3; C4, complement C4; anti-dsDNA, anti-double stranded DNA antibody; ANA, antinuclear antibodies; SLE-DAI, systemic lupus erythematosus disease activity index. *P* < 0.05, *P* < 0.01, *P* < 0.001 (Mann-Whitney U test).

### Urine sample collection and 16S rRNA gene sequencing

In this study, the morning urine samples (10 mL) was collected from hospitalized SLE patients. Each enrolled patient was instructed and required to provide a clean-catch midstream urine sample. All urine samples were assigned a unique identification code and promptly transferred to the laboratory. The samples were subjected to centrifugation (4, 000 g/15 min/4°C) to separate the pellet from the supernatant. After collecting the pellet, it was transferred into a sterile 2 mL centrifugation tube and stored at -80°C until needed for DNA extraction by the MagAttract PowerSoil Pro DNA Kit. To comprehend the DNA quality and quantity used 1% agarose gel. Sterile water was used for DNA dilution (1 ug/L) depending on the concentration. A particular primer (338F:ACTCCTACGGGAGGCAGCAG; 806R:GGACTACHVGGGTWTCTAAT) with the bar code was utilized to amplify the 16S genes of the different areas (V3-V4). Each PCR reactions had the following ingredients: 4 μL 5FastPfu Buffer, 2 μL 2.5 mM dNTPs, 0.8 μL Forward Primer (5 M), 0.8 μL Reverse Primer (5 M), 0.4 μL FastPfu Polymerase, 0.2 μL BSA, 10 ng template DNA, and ultimately 20 μL ddH2O. Thermal cycling included an initial denaturation step (95°C/3 min), followed by 29 cycles of denaturation (95°C/30 sec), annealing(53°C/30 sec) and elongation (72°C/10 min) and set to 10°C till opened. Equal density proportions were used to merge the PCR products. To purify the PCR product mixture, a Qiagen Gel Extraction Kit (Qiagen, Germany) was employed. TruSeq^®^ DNA PCR-Free Sample Preparation Kit (Illumina, USA) was utilized to generate sequencing libraries, index codes were added as per manufacturer’s instructions. The quality of the library was assessed using the Qubit@2.0 Fluorometer (ThermoFisher Scientific) and the Agilent 2100 Bioanalyzer platform. Subsequently, the library was sequenced on an Illumina Miseq instrument, generating paired-end reads with a length of 300 bp. NCBI’s Sequence Read Archive (SRA) database holds the raw data reads deposit with the Accession Code: PRJNA961838.

### Processing, analysis, and annotation of sequencing data for taxonomic classification

We applied a preliminary filtering process to the raw data to verify its quality and precision. Sequences that fell outside the length range of 200-550 base pair, included uncertain bases, had a poor-quality rating (≤ 20), or did not entirely match the primer and bar code sequences were excluded from further analysis sample-specific barcode sequences were used to separate the remaining high-quality sequences. The Unoise3 technique in usearch11 was then employed to de-noise the high-quality sequences. Sequences that have been denoised via Unoise3 are usually called amplicon sequence variants (ASVs). All sequences were categorized into several taxonomic categories using the BLAST program in comparison to the SILVA138 database.

### Bioinformatics and statistical analysis

Rarefaction curves were generated using QIIME (v1.8.0) to evaluate sequencing depth and sample richness. Various metrics, including ACE, Chao1, richness, goods_Coverage, PD_whole_tree, Shannon, and Simpson, were used to assess bacterial diversity and abundance in urine samples. α diversity (ASVs in QIIME) and β diversity (PCoA) were analyzed to examine bacterial composition variation. The Wilcoxon rank test identified distinct species or traits, while the LEfSe technique (LDA effect size) detected species with significant abundance changes across groups. Statistical analysis was performed using SPSS 24.0 and GraphPad Prism 8.0, with a significance level set at *P* < 0.05. AUC for each prediction model was calculated using SPSS after constructing ROC curves. Random forest analysis was employed to create inter-group prediction models, and the “Mean Decrease Accuracy” was calculated via “Random Forest” package in the R language. We confirmed the optimal modeling combination by using 5-fold cross-validation has been tested in five trials. Model calibration was performed using the “caret” package. The AUC value was calculated and the “pROC” tool was employed to plot the ROC curve. The functional prediction analysis was performed by the Phylogenetic Investigation of Communities by Reconstruction of Unobserved States (PICRUSt) method to determine Kyoto Encyclopedia of Genes and Genomes (KEGG) pathways.

## Results

### Comparative analysis of α and β diversity in the urinary microbiome

**Figure 2:**
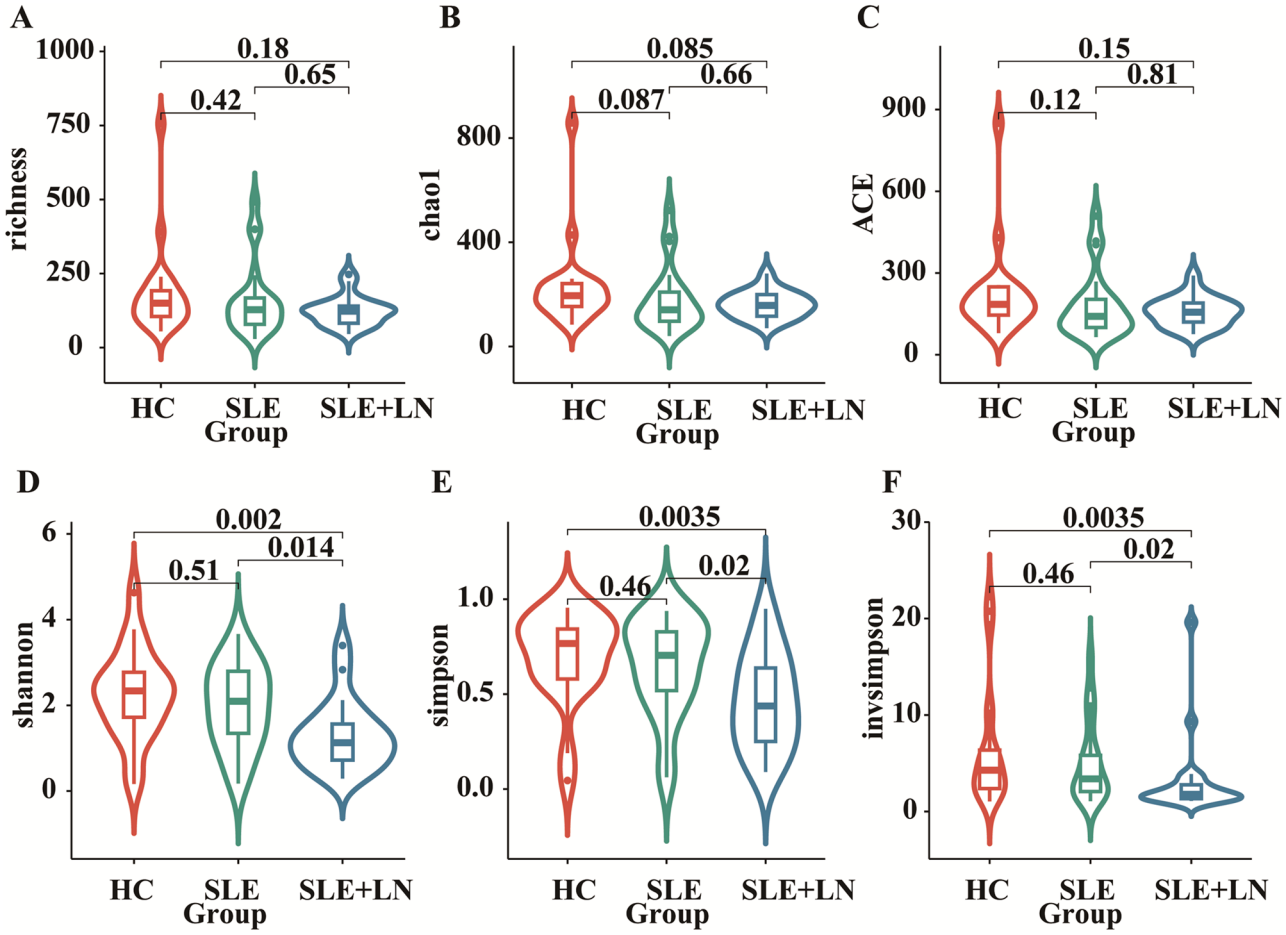
Comparison of urinary bacterial diversity among SLE with LN, SLE without LN, and HC Groups: Alpha Diversity Metrics. ACE **(A)**, Chao1 **(B)**, goods_coverage **(C)**, PD_whole_tree **(D)**, Shannon **(E)**, and Simpson **(F)**.

**Figure 3:**
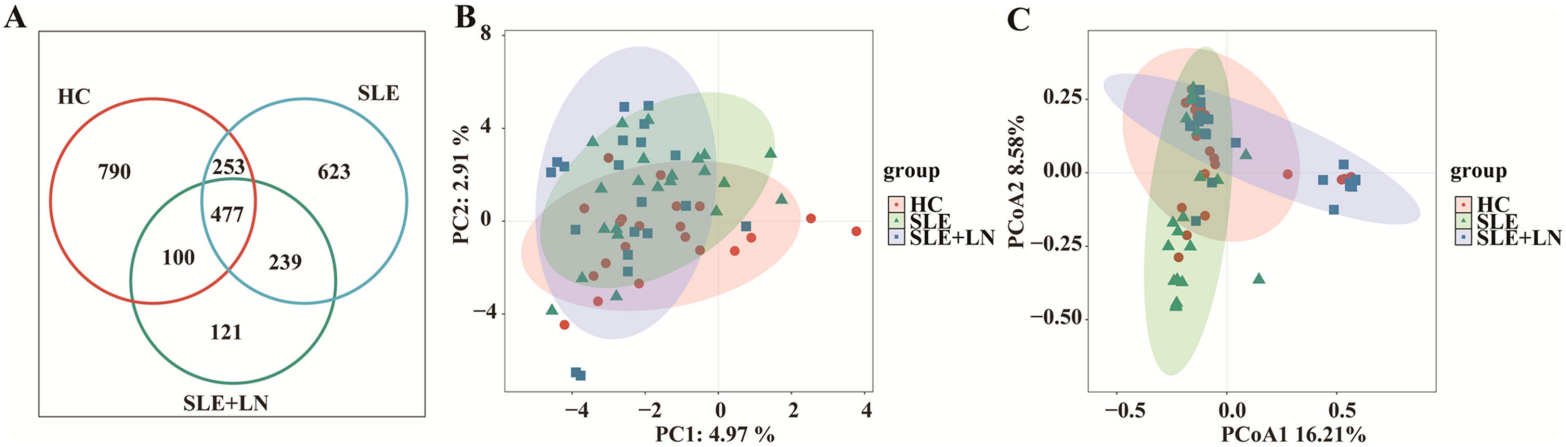
Changes of the composition of urinary bacteria. **(A)** Venn diagram analysis according to the amplicon sequence variants abundance among the three groups. **(B)** Constrained Principal coordinate analysis of Bray–Curtis distance with each sample colored according to different groups. **(C)** Non-metric multidimensional scaling (NMDS) analysis of each sample colored according to different groups. HC, healthy control; SLE, SLE without LN; LN, SLE with LN.

To investigate whether there are changes in microbial diversity in the urine of SLE patients, we initially conducted 16S rRNA sequencing analysis on the microbial communities present in the urine sediment of SLE patients, LN patients, and healthy controls. The saturation curves provided evidence that the amount of sequencing data available was sufficient for performing analyses at the level of individual species. Afterward, we conducted an analysis of the α diversity of the bacterial community in each group using six different indices: ACE, Chao1, goods coverage, PD_whole_tree, Shannon, and Simpson. We found that the Shannon index, Simpson diversity index, invsimpson diversity index which measures α diversity, showed a significant decrease in bacterial community diversity in SLE patients with LN group, when compared to SLE patients without LN group or HC group (**Figures 2D–F**). Indicators of α-diversity showed a decreasing trend in the SLE group compared to the healthy group; however, these differences were not statistically significant (**Figures 2A–F**). The Venn diagram indicates that there are 477 ASVs shared among three groups. Additionally, HCs, SLE and LN groups had 790, 623, and 121 unique ASVs, respectively (**Figure 3A**). To investigate the β diversity in urinary microbial communities across these groups, we utilized PCA and PCoA analysis (**Figures 3B, C**) which demonstrated clear distinctions between the HCs and SLE individuals with or without LN. In summary, these results indicate that SLE patients exhibit a distinct bacterial profile from HCs.

### Taxonomic abundance analysis of the urinary microbiome

**Figure 4:**
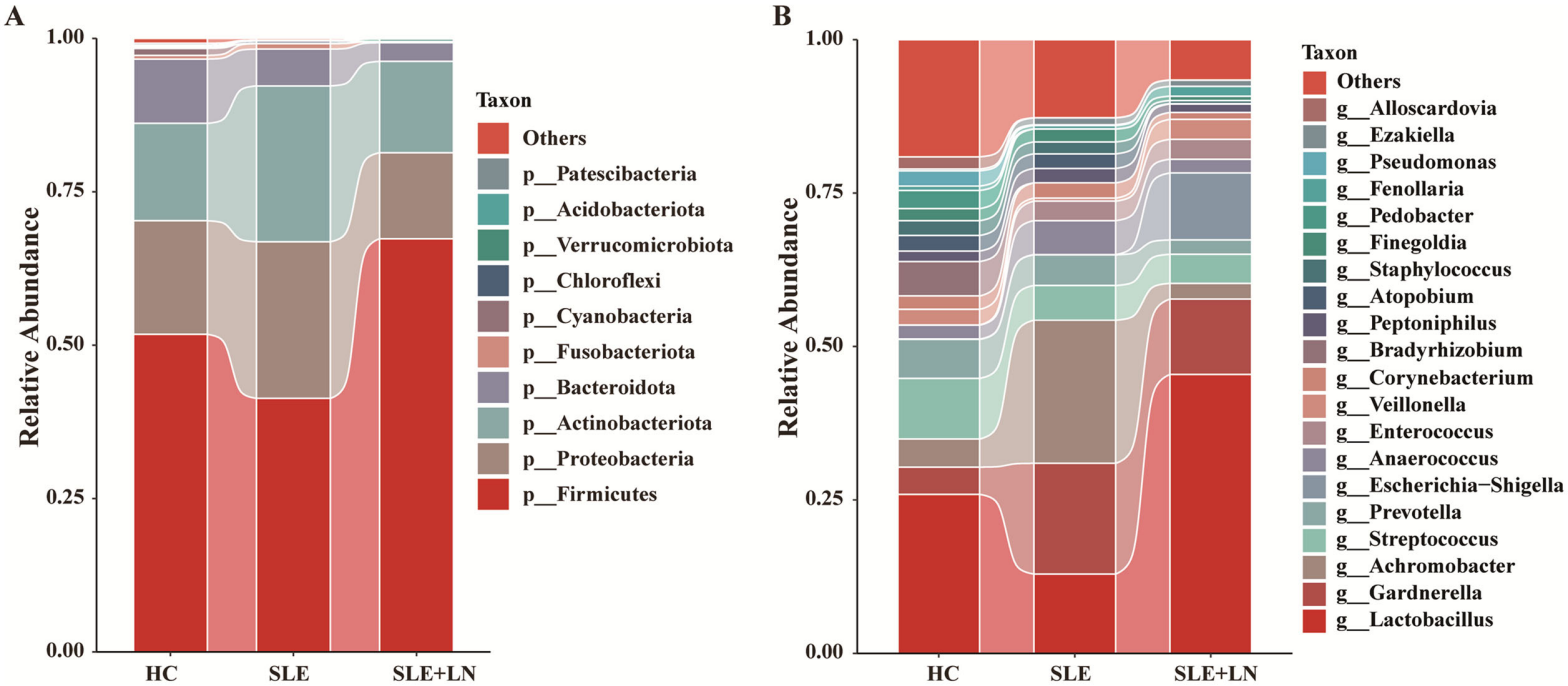
Diversity of the composition of urinary bacteria. **(A)** Comparisons of the relative abundance of dominant bacteria taxa at the phylum level among groups. **(B)** Comparisons of the relative abundance of dominant bacteria taxa at the genus among groups.

**Figure 5:**
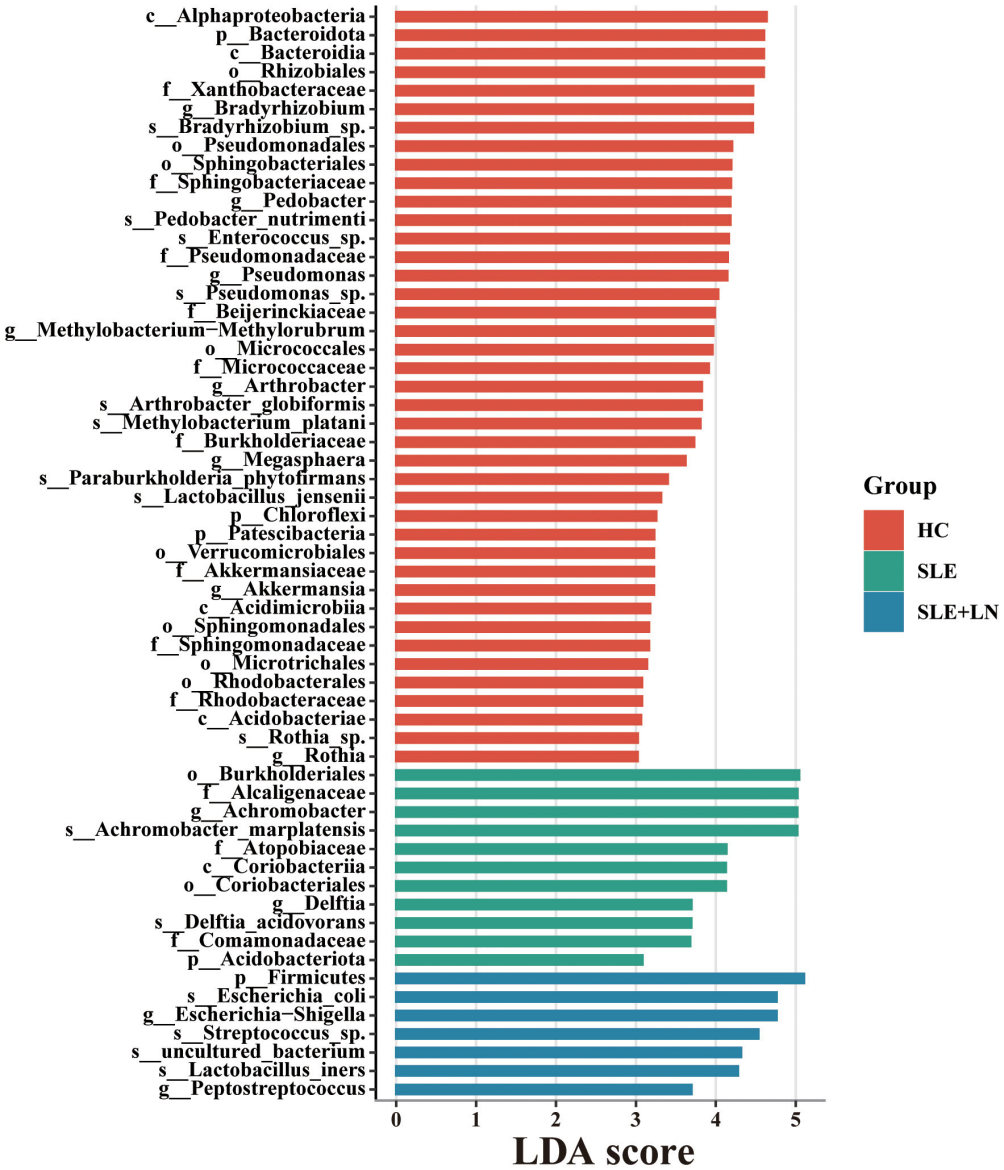
LDA score computed by LEfSe analysis among three groups (LDA score >3, red, HC; green, SLE without LN; blue, SLE with LN).

**Figure 6:**
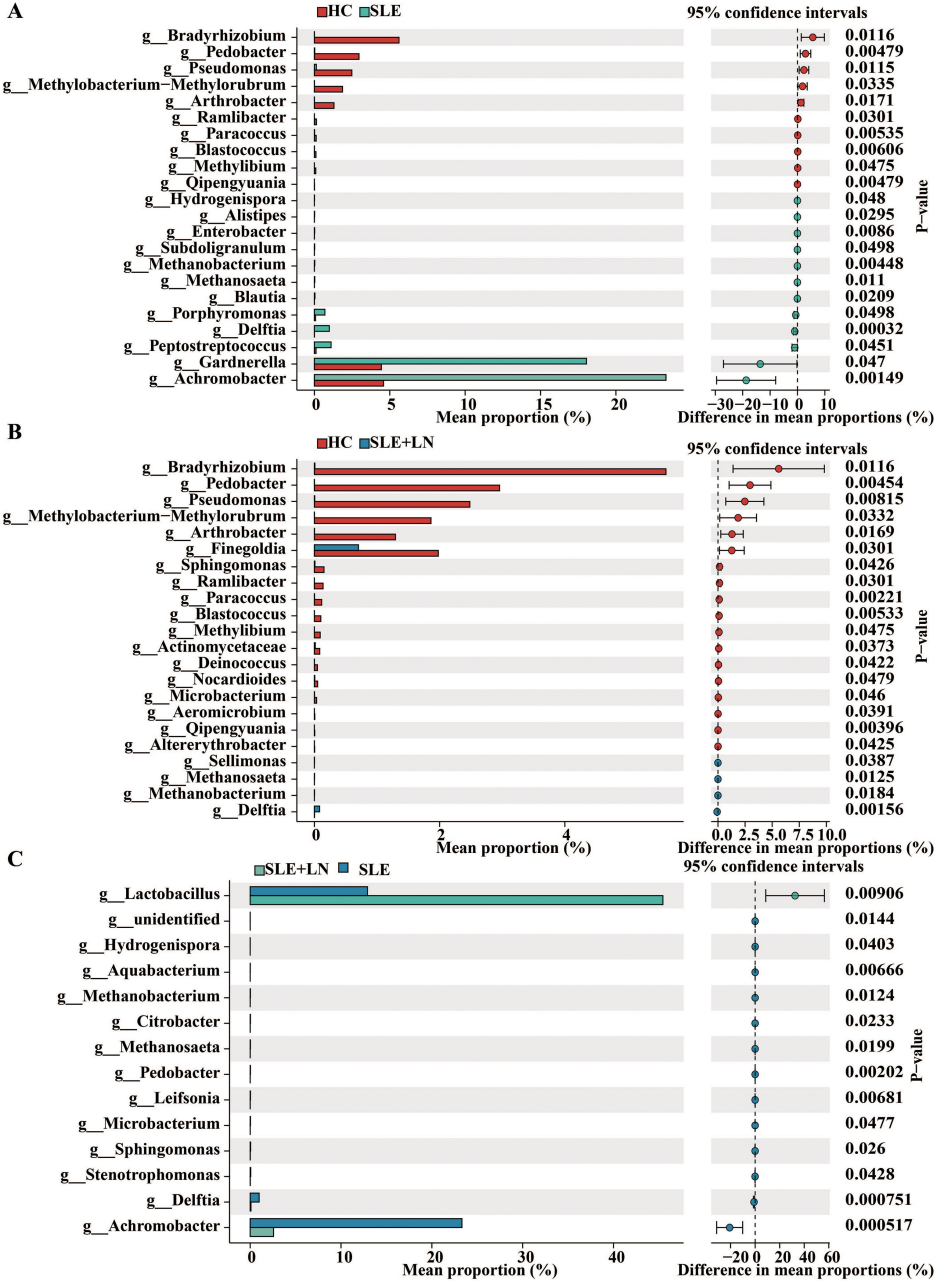
STAMP (statistical analysis of taxonomic and functional profiles) Analysis of Urinary Bacteria Microbiota. **(A)** STAMP analysis of urinary bacteria at the phylum level between HC and SLE group. **(B)** STAMP analysis of urinary bacteria at the phylum level between HC and SLE with LN group. **(C)** STAMP analysis of urinary bacteria at the phylum level between SLE and SLE with LN group.

The microbiota plays a crucial role in the pathogenesis of autoimmune diseases. To investigate the attributes of urine’s microbiome in SLE, we analyzed the taxonomic organization and relative abundance of the microbiota in the urine sediment of SLE patients with or without LN and HCs at various taxonomic levels. At the phylum level, *Firmicutes*, *Proteobacteria*, and *Actinobacteria* dominate in absolute abundance, consistent with previous studies on the human urinary microbiota (**Figure 4A**). When examining the genus level, *Gardnerella* showed high abundance in SLE patients with or without LN compared with HCs. Furthermore, in LN patient’s urinary microbiome *Lactobacillus* in LN patients were more abundant (**Figure 4B**). To further analyze the differences in urinary microbiota among the groups, we utilized LefSe analysis, and detected eight distinct taxa, including classes, species, phyla, orders, families, and genera, as potential markers across all group comparisons. Additionally, the Cladogram displayed these differential taxa (**Figure 5**). Furthermore, the analysis of urinary microbiota using STAMP proved that genus which was a statistically significant factor in the disease group compared with HC group (**Figures 6A–C**) (*P* < 0.01).

### Development of a diagnostic model for SLE using urine microbiota

**Figure 7:**
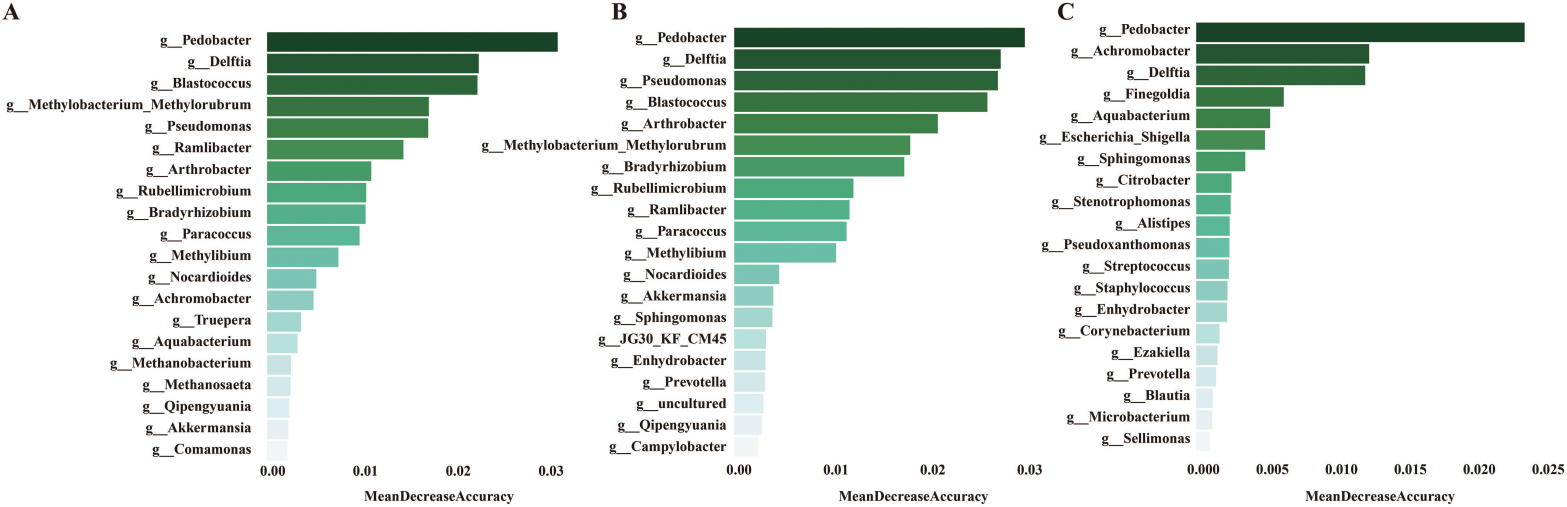
**(A)** Mean decrease accuracy of top 20 bacteria screened by lefse between HC and SLE group. **(B)** Mean decrease accuracy of top 20 bacteria screened by lefse between HC and SLE with LN group. **(C)** Mean decrease accuracy of top 20 bacteria screened by lefse between SLE and SLE with LN group.

**Figure 8:**
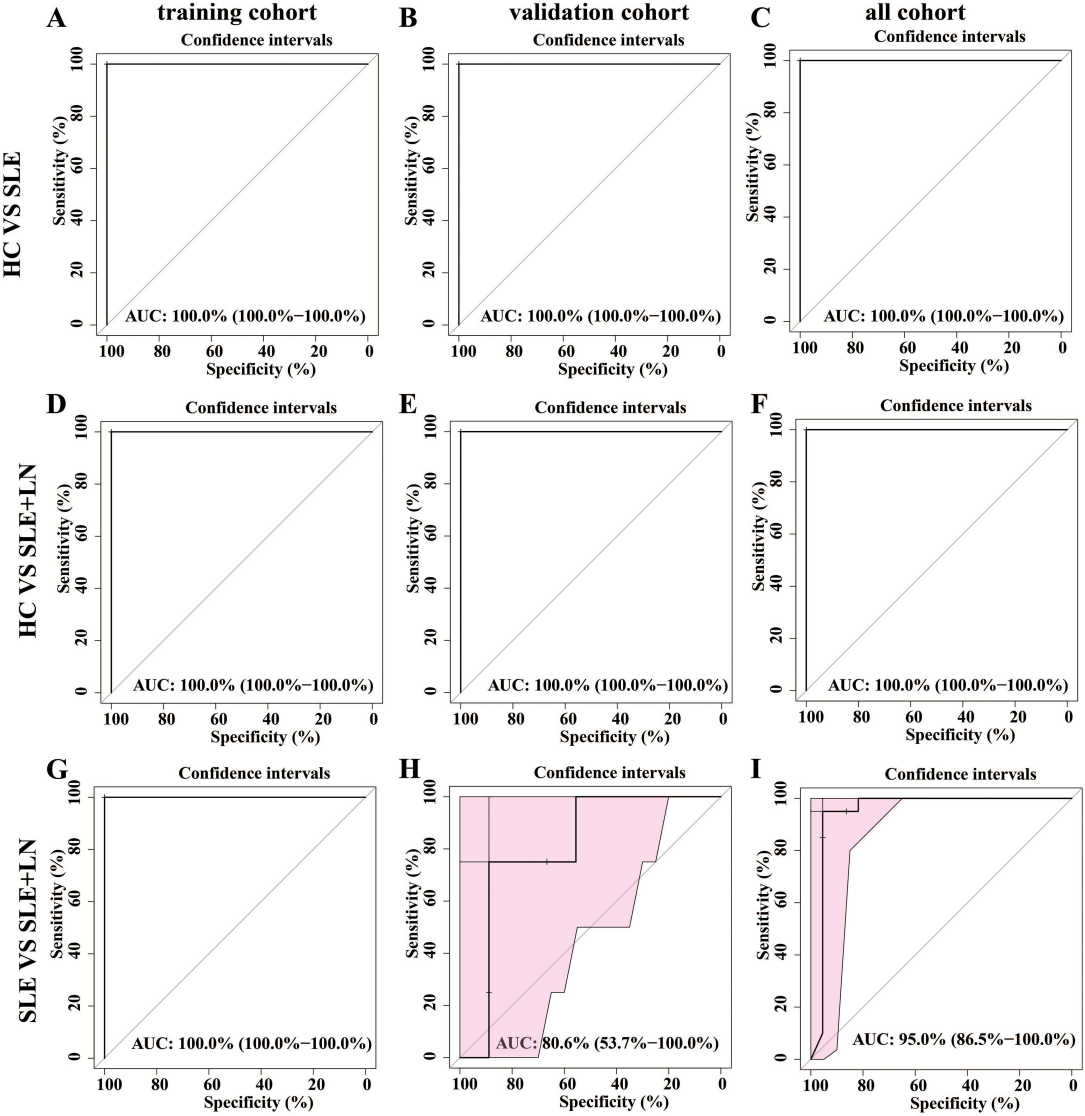
Establishment of a predictive classifier based on the gut microbiome profiles. **(A–C)**. The ROC curve for the training cohort/validation cohort/all microbiome profiles based on the random forest classifier between HC and SLE group. **(D–F)**. The ROC curve for the training cohort/validation cohort/all microbiome profiles based on the random forest classifier between HC and LN group. **(G–I)** The ROC curve for the training cohort/validation cohort/all microbiome profiles based on the random forest classifier between SLE and LN group.

To develop a diagnostic model for SLE based on urine microbiota, we employed a random forest algorithm to analyze all genus-level bacteria, identifying the top 20 genera that differentiate among the three groups. (**Figures 7A–C**). Subsequently, we conducted modeling analyses on the top 5 genera ranked by importance among the three groups. The model was trained with randomly selected 70% samples and tested on the remaining 30% samples. The model successfully distinguished among the three groups in all pairwise comparison using the distinct pattern of urinary microbiome markers (**Figures 8A–I**). The diagnostic model achieved a perfect area under the curve (AUC) of 100% between SLE versus HC. Similarly, wo also develop a diagnostic model consist of 18 bacterial markers and achieved an AUC of 100% between LN versus HC.

### Correlation between urinary microbiome and clinical parameters in SLE patients

**Figure 9:**
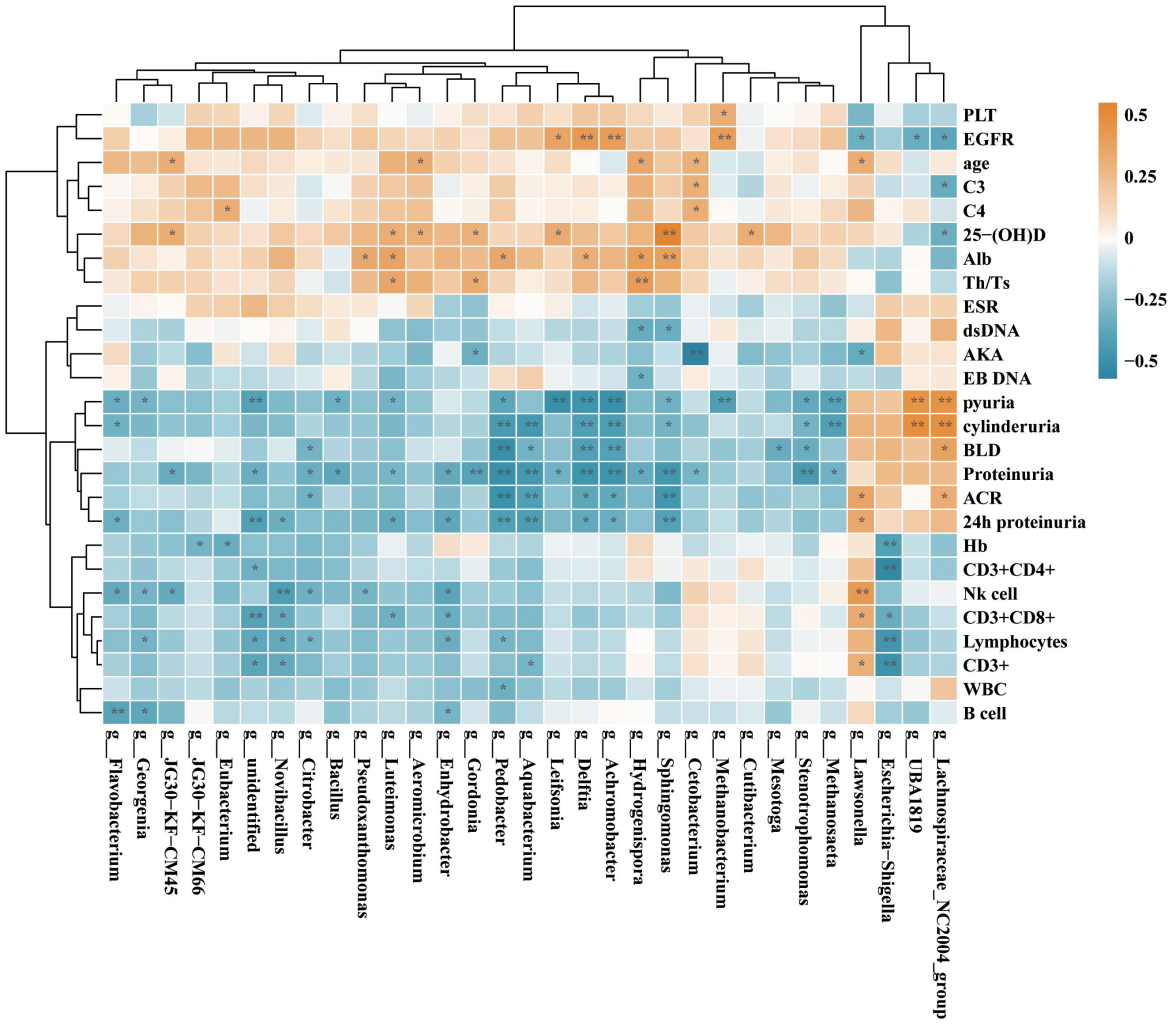
Correlation analysis of the differential genera and clinical parameters between SLE and SLE with LN group according to the Spearman’s correlation analysis. The correlation effect is indicated by a color gradient from blue (negative correlation) to orange (positive correlation). Correlation coefficients and *p*-values (*p < 0.05; **p < 0.01) are shown.

To explore the connection of urinary microbiome with clinical parameters attributes of SLE patients, we conducted a comprehensive analysis of the abundance of the top 30 differentially enriched species in SLE patients with or without LN. The strength of correlation was represented by a color gradient, ranging from negative (blue) to positive (orange). Our findings demonstrated significant positive correlations between *Sphingomonas*, *Lachnospiraceae* genera in SLE patients with parameters such as vitamin D, cylinderuria, and proteinuria. Furthermore, *Pedobacter*, *Aquabacterium*, *Delftia*, *Achromobacter* displayed a negative correlation with proteinuria proteinuria and ACR (**Figure 9**). This suggests that alterations in urinary microbiota may be involved in the progression of LN.

### Functional pathways influenced by microbial community in SLE patients and HCs

**Figure 10:**
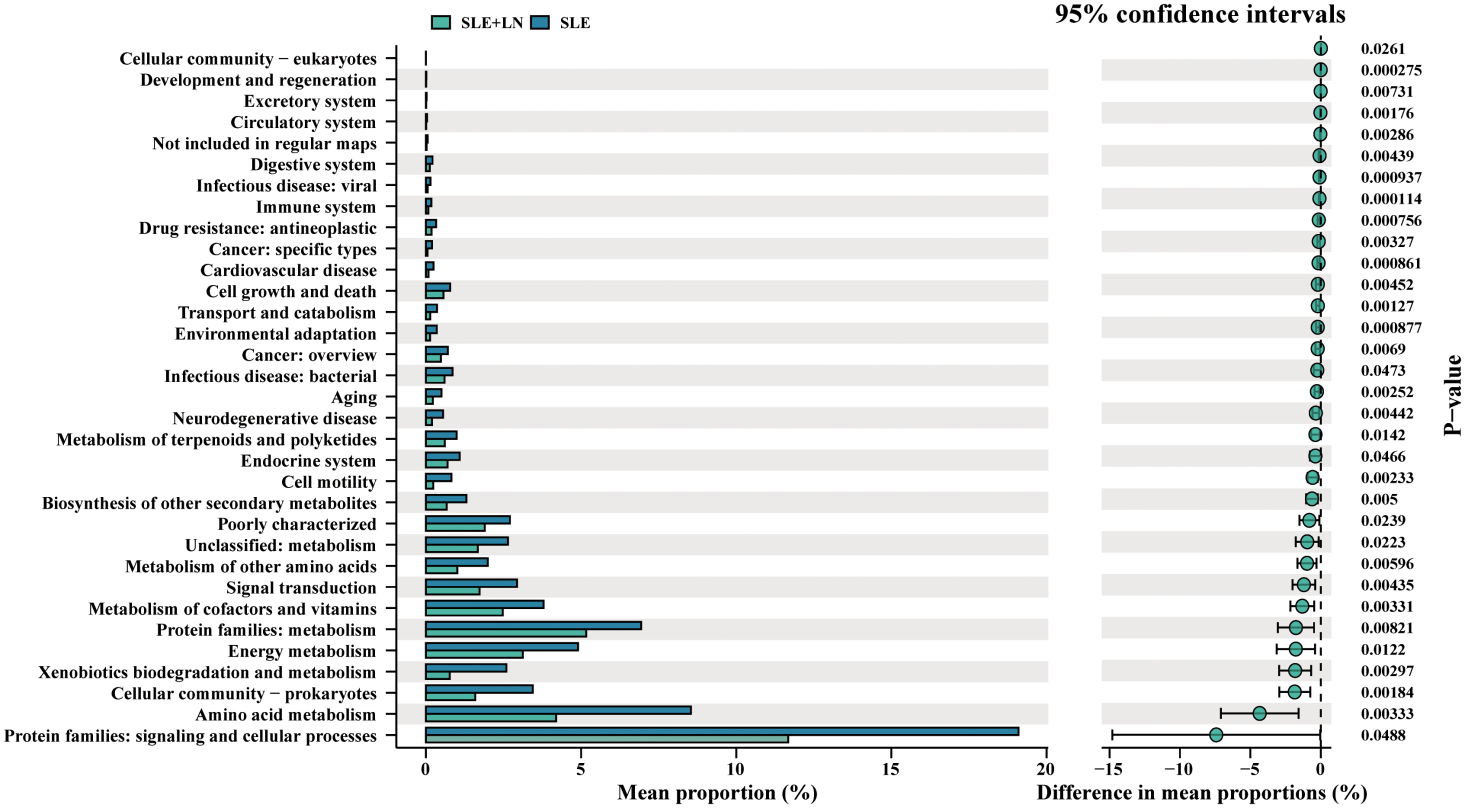
Bacteria function classification prediction between SLE with and without LN groups of t-test statistical tests.

Existence of a healthy microbial ecosystem on the urine is crucial for its overall health and maintenance (Chen et al., 2022). To explore the potential involvement of the urine microbiome, we employed PICRUSt analysis, utilizing the KEGG database, to predict microbiota-associated functional pathways that exhibited differing abundances between SLE with LN and without LN patients. The predicted KEGG pathways significantly enriched in Lupus nephritis included protein families: signaling and cellular processes, amino acid metabolism, xenobiotics biodegradation and metabolism, metabolism of cofactors and vitamins compared with that of SLE without LN group (**Figure 10**). There have been reports in the literature documenting the relationship between alterations in metabolic pathways and the pathogenesis of SLE. The changes in predicted capability may suggest that composition of the urine microbiome may influence LN progression. Together, taxonomic profile encoded by urine bacteria represents differential enrichment of predicted functional pathways, which are associated with clinical features of LN.

## Discussion

SLE is an autoimmune disease characterized by the involvement of one or more organs, including the skin, kidneys, joints, and nervous system, and take a chronic or relapsing and remitting disease course (Zimlichman et al., 2014; Durcan et al., 2019; Parikh et al., 2020; Tsokos, 2020; Lou et al., 2022). There are six distinct histological groups of LN that are distinguished from one another by the severity of their involvement with the kidneys in LN and their specific symptoms and varied treatment approaches (Anders et al., 2020). Over the past few decades, immunosuppressive medication, commonly with mycophenolate mofetil or cyclophosphamide and glucocorticoids, is the conventional course of action for LN. Despite advancements in treatment and diagnostic option, LN is still considered the most lethal for SLE patients (Morales et al., 2021). The numerous comorbidities linked to immunosuppressive therapy, such as infections, osteoporosis, cardiovascular consequences, and reproductive issues, are alarming (Aziz and Chaudhary, 2018; Yu et al., 2022). Rapid and precise LN diagnosis and prompt start of treatment can be vitally beneficial for improving the ultimate outcomes in SLE patients. In this work, urine microbiomes of three groups (HCs, SLE with LN and SLE without LN) were performed by16S rRNA sequencing. We have further developed a diagnostic model consisting of a panel of different bacterial in identifying LN from SLE patients. This research provides a new avenue of research focusing on the changes of urinary microbiome in SLE patients and establish connections between specific urinary genera and clinical parameters.

Infections are considered important environmental triggers of autoimmunity and can contribute to autoimmune disease onset and severity (Qiu et al., 2019). Mirei Shirakashi et al. found that defective T cell receptor (TCR) signaling alters the gut microbiome and promotes systemic autoimmunity by driving the differentiation of Th17 cells (Shirakashi et al., 2022). Seung-Chul Choi et al. discovered that gut microbiota dysbiosis and altered tryptophan catabolism contribute to autoimmunity in lupus-susceptible mice (Choi et al., 2020). However, the etiologic mechanism between urinary microbiota and the occurrence of LN in SLE patients remains not fully understood. There is emerging proof that the pathophysiology of numerous autoimmune illnesses is influenced by abnormalities in the gut microbial flora (Rosser and Mauri, 2016) including type 1 diabetes (Gulden et al., 2018), rheumatoid arthritis (Zhang et al., 2015), multiple sclerosis (Cignarella et al., 2018), inflammatory bowel disease (Schirmer et al., 2019) and SLE (Silverman, 2019; Tomofuji et al., 2021; Chen et al., 2022). It is found that *Ruminococcus gnavus* (RG) intestinal expansions and overall disease activity has direct correlation primarily in LN patients (Azzouz et al., 2019). However, there is limited research investigating the association between renal and urinary microbiota involvement in SLE. Pachucki RJ et al. find that persistent bacteriuria and antibodies recognizing curli/edna complexes from *Escherichia coli* are linked to flares in systemic lupus erythematosus (Pachucki et al., 2020). For many years, a urologist’s concern with urine bacteria culture was limited to making a diagnosis of a urinary tract infection. However, a research investigation involving 77 catheterized patients revealed the presence of 78 unique bacterial species in their urinary tracts, thus challenging the previously held notion of sterility within the urinary tract (Thomas-White et al., 2018). Besides, our research (Yang et al., 2022) and others (Nejman et al., 2020; Narunsky-Haziza et al., 2022) have also revealed that the bacteria and fungi within tumors were located in both immune and malignant cells and that the composition varied according to tumor type and this awareness could also be extended to the bladder.

Thus, this work showed that SLE patients with LN and without LN exhibited significant variations in the bacterial profiles of their urine compared to the HC group. In comparison to the SLE group, notable disparities were observed in the α diversity of bacterial markers specifically within SLE patients with LN, as evidenced by variations in the Shannon and Simpson indices. These important findings offer insights into how the microbiome of urinary system may be involved in the SLE progression. It also lay the foundation for further research on understanding the mechanisms behind urinary microbiota dysbiosis in SLE patients. *Streptococcus* and *Prevotella* showed decreased abundance in SLE patients with and without LN than its abundance in HCs. Notably, *Gardnerella* which is commonly associated with vaginitis has high abundance in the urine bacteria of SLE patients which consistent with the previously reported trend of the gut microbiota and to be proved negatively correlated to the *Lactobacillus* abundance in their vagina (Ling et al., 2023). These results imply potential interactions between that vaginal and urinary microbiota. Research efforts directed at exploring the connections between these two microbiomes can be of high value. Of particular interest, our study reveals that *Sphingomonas*, *Lachnospiraceae* are positively correlated with vitamin D, cylinderuria, and proteinuria while *Pedobacter*, *Aquabacterium*, *Delftia*, *Achromobacter* displayed a negative correlation with proteinuria and ACR. Vitamin D receptor ligands can mediate immunosuppressive effects. Studies have reported that vitamin D may play a regulatory role in the interferon alpha amplification loop in SLE (Ben-Zvi et al., 2010). Therefore, we propose that the microbiome may mediate vitamin D receptor regulation of interferon alpha, contributing to the exacerbation of SLE. Moreover, administration of *Lactobacillus* spp. to mice with LNs can influence its progression by enhancing immune regulation, providing protection against vascular diseases, or exerting anti-inflammatory effects. Therefore, *Lactobacillus* appearance in urine of LN patients may potentially highlight a protective effect. In addition, functional prediction of unique bacteria using Picrust2 and KEGG analysis showed that these unique bacteria may regulate protein families involved in signaling and cellular processes, vitamins and cofactors metabolism, indicated to influence the SLE progression.

The dysbiosis within the gut (Zheng et al., 2023), subgingival (Correa et al., 2017), cutaneous (Huang et al., 2020), oral (van der Meulen et al., 2019) and vaginal (Ling et al., 2023) microbiomes of SLE patients have been implicated in contributing to immune dysregulation. For the first time, we observed a correlation between urinary microbiota and disease immune markers in SLE patients. However, we must acknowledge the limited size of our single-center urine samples and the large percentage of unidentified taxa are two factors that restrict our existing data. Additionally, further exploration of how microbiota-modulating therapies, such as probiotics and antibiotics, may influence disease progression in SLE patients is a valuable direction for future research. Also, diagnostic markers in the urine samples for LN, using only bacterial genera, which is a big limitation for the data. To address these limitations in future studies, we will recommend implementing longitudinal designs to track changes over time and employing metagenomic analyses to capture the complexity of the microbiome. These approaches could significantly enhance the validity of our findings and provide deeper insights into the interactions between microbial communities and SLE. Lastly, we did not conduct functional validation and mechanistic studies to exclude potential influences from fungi or viruses among the differing microbial taxa, which necessitates further investigation in future research. In summary, our research is the first to indicate the potential use of urinary bacterial markers for predicting LN by outlining the variation in urine mycobiome equilibrium between the groups with and without LN in SLE.

## Data Availability

The datasets presented in this study can be found in online repositories. The names of the repository/repositories and accession number(s) can be found in the article/supplementary material.
